# Mothers’ pupillary responses to infant facial expressions

**DOI:** 10.1186/s12993-017-0120-9

**Published:** 2017-02-06

**Authors:** Santeri Yrttiaho, Dana Niehaus, Eileen Thomas, Jukka M. Leppänen

**Affiliations:** 1 Tampere Center for Child Health Research, School of Medicine, University of Tampere, Lääkärinkatu 1, 33520 Tampere, Finland; 2 Department of Psychiatry, Faculty of Health Sciences, Stellenbosch University, Stellenbosch, South Africa

**Keywords:** Pupil, Emotion, Facial expressions, Attention, Mothers, Infant faces

## Abstract

**Background:**

Human parental care relies heavily on the ability to monitor and respond to a child’s affective states. The current study examined pupil diameter as a potential physiological index of mothers’ affective response to infant facial expressions.

**Methods:**

Pupillary time-series were measured from 86 mothers of young infants in response to an array of photographic infant faces falling into four emotive categories based on valence (positive vs. negative) and arousal (mild vs. strong).

**Results:**

Pupil dilation was highly sensitive to the valence of facial expressions, being larger for negative vs. positive facial expressions. A separate control experiment with luminance-matched non-face stimuli indicated that the valence effect was specific to facial expressions and cannot be explained by luminance confounds. Pupil response was not sensitive to the arousal level of facial expressions.

**Conclusions:**

The results show the feasibility of using pupil diameter as a marker of mothers’ affective responses to ecologically valid infant stimuli and point to a particularly prompt maternal response to infant distress cues.

**Electronic supplementary material:**

The online version of this article (doi:10.1186/s12993-017-0120-9) contains supplementary material, which is available to authorized users.

## Background

Parental care and parent-infant interaction relies heavily on the ability to receive and express nonverbal emotional signals through facial expressions [1]. There is increasing interest in the neurocognitive bases of these capacities in parents [2–4] and infants [5], and in the possibility that subtle variations in emotional signaling may have important influences on the quality of parent–child attachment [6]. In the current study, we extend these studies by examining whether mothers’ pupil dilation is sensitive to children’s affective cues and could, in future, serve as an accessible marker of interindividual variation in these responses. If the pupil is sensitive to mothers’ affective responses, such as heightened vigilance towards infant signals of discomfort and distress [1], this index may prove useful in studying and understanding the mechanisms underlying maternal sensitivity or neglect.

To begin examining whether pupil diameter is a sensitive index of parents’ physiological responses to children’s affective cues, the present study focused on mothers’ responses to infant facial expressions. Despite increasing involvement of fathers in childcare in many societies and the need for research on biological bases of paternal childcare, there are known intersex differences in the neural and hormonal bases for caregiving behaviors [3]. For this reason, we limited our current investigation on parental responsiveness to infant emotion to mothers rather than sampling parents of both sexes. The neural and physiological basis of mothering constitutes a distinct domain of research [7], with potentially important implications for maternal and infant mental health. Previous studies show differential patterns of brain and behavioral responses elicited by infant as opposed to adult faces [8–11], especially in women [11]. Further, mothers, compared to nulliparous women, show more marked early frontal (∼100 ms) event-related potentials to infant facial expressions, as well as more pronounced modulation of posterior visual responses to infant faces displaying negative emotions [2, 12]. The current study extends the research on neurophysiological processes of mothering by examining the pupillary correlates of mothers’ responses to infants’ positive and negative facial expressions [12, 13].

Pupil size is largely determined by reflexive control over light entering the eye [14]. However, pupil diameter is also influenced by the activity of the sympathetic autonomic nervous system (ANS) during emotional arousal [15, 16]. The pupillary response to a visual stimulus can typically be characterized by two consecutive phases. First, in response to increased brightness, there is a constriction in pupil size around 600–1600 ms after stimulus onset [15]. Following the constriction, the pupil starts to dilate back to a baseline level over the course of several seconds. For example, an initial constriction of the pupil in response to visual stimuli is followed by a slow dilation that is augmented for emotionally positive and negative scenes [15, 17]. Pupil constriction and dilation per se are brought about by distinct branches of the nervous system, parasympathetic and sympathetic, innervating the constrictor and dilator muscles, respectively [16]. Pupil size at any time reflects the tone of both of these muscles. Therefore, both the constriction and the dilation phase are susceptible to emotional effects elicited by emotionally arousing scenes and facial expressions [15, 17–19]. Pupil response to emotional factors is thought to be mediated by the modulatory effects of the brain’s noradrenergic system on neural circuitry controlling the muscles of the iris [20, 21]. Importantly, larger pupil dilation to emotional stimuli cannot be suppressed voluntarily [22], making it an accessible marker for studies examining human affective responses in a variety of contexts.

Because the dominant source of variability in pupil size comes simply from changes in stimulus luminance [20], pupillometry studies have traditionally required stringent control of luminance levels across experimental stimuli or, at minimum, equalization of mean luminance across different stimulus categories. While the luminance of many types of visual stimuli can, in principle, be easily adjusted to equal mean level, such equalization is not viable for all studies and small deviations from the mean levels will be unavoidable. This poses particular challenges for studies examining pupillary responses to ecologically valid stimuli such as infant facial expressions. The only available method to capture preverbal infants’ facial expressions is to photograph them as they occur spontaneously in variable environments where strict control of luminance is difficult or may result in unnatural quality of the stimuli. Corresponding problems exists in other contexts such as studies using facial stimuli presented in live face-to-face or over-the-internet conditions, faces in variable background illumination, and faces of people with variable ethnicity or hair color. For this reason, an important challenge for pupillometric studies is to examine ways to disentangle unavoidable pupillary responses to luminance changes from those involved in affective processing.

The current study consisted of two experiments where pupil response was measured in response to infant facial stimuli. In Experiment 1, a large sample of mothers of young infants (*N* = 86) was recruited in order to examine whether pupil constriction and dilation are sensitive physiological measures of mothers’ responses to mild and strong instances of infant positive and negative affect. Based on prior studies [15–20], we predicted relatively larger pupil diameter in response to high-arousal facial expressions (i.e., high intensity positive and negative expressions) in both the constriction and dilation phases. Our secondary aim was to disentangle affective pupillary responses to facial expressions from the response to unavoidable variations in stimulus luminance. To this end, we carried out Experiment 2, a control experiment, where participants were presented both with faces (face condition) and luminance-matched non-face stimuli (control condition). We hypothesized that an emotive pupil response, a greater pupil diameter during pupil dilation and constriction phases, would be manifested in the face condition but not in the control condition.

## Experiment 1

### Methods

#### Participants

The participants were mothers of young infants participating in an ongoing longitudinal study examining the mental health of mother-infant dyads in the Cape Town metropolitan area, South Africa. The participants had no mental health disorder as assessed by a psychiatrist through clinical assessment and the Mini International Neuropsychiatric Interview [23]. The eye-tracking assessment in mothers was conducted as a part of a scheduled immunization visit to a private well-baby clinic at the infant age of 6 weeks. All mothers recruited to this study and tested by the eye-tracking procedure by September 30th, 2015, were included in the current analyses. The final sample consisted of 86 mothers (Age: *M* = 32.4 years, SD = 5.4 years) of Caucasian (*N* = 53) and Black (*N* = 33) ethnicity. The Caucasian participants had higher socioeconomic status (SES) than the Black participants as indexed by monthly income [*Χ* ^2^(3) = 57.91, *p* < .001], level of education [*Χ* ^2^ (2) = 66.32, *p* < .001] and employment [*Χ* ^2^ (3) = 66.89, *p* < .001]. The number of pregnancies of the participants ranged from 1 to 5 with 68.7% of participants having more than one pregnancy.

#### Stimuli

**Fig. 1:**
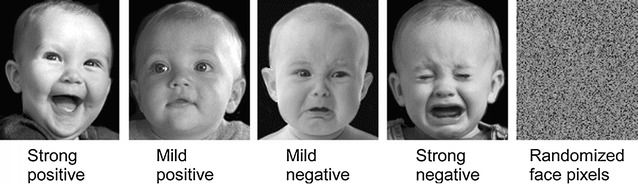
Examples of infant face stimuli from each stimulus category defined by the intensity and valence of the facial expression of emotion. Randomized pixels (on the *right*), derived from all face stimuli were used as a pre-stimulus display. In the control experiment (Experiment 2) randomized face pixels, derived from each face stimulus individually, were used in place of the face stimuli in the “non-face” condition

The infant face stimuli were obtained from an existing stimulus set [13]. These 36 grayscale photographs were close-ups of infants with black uniform backgrounds, with infants producing four types of facial expressions (Fig. 1): mild positive (MP), strong positive (SP), mild negative (MN), and strong negative (SN). It is noteworthy that this classification is based on previous work showing that infants’ emotional expressions during the first year of life are not readily categorized into discrete emotion categories, but instead, to primary dimensions of hedonic pleasure and arousal [13]. Nine instances of each category were used in the experiment. The selection was made to obtain sets of images that were matched for face-background ratio, models’ head orientation, and models’ age as closely as possible. As the original pool of images was taken from separate sources, some variations in above characteristics remained in each stimulus category. The infant’s eyes were open in all positively-valenced (MP and SP) photographs but were closed in 67% of images with strong (SN) and in 22% of images with mild negative (MN) facial expressions. The infants had open eyes with direct gaze in 33, 56, 11, and in 33% of pictures classified as SP, MP, SN, and MN, respectively. All infants depicted in the stimuli were Caucasian.

The current infant stimuli were selected from an original pool of 208 images consistently rated (>75%) as reflecting one of the four emotions (i.e., MP, SP, MN, or SN) by human judges [12, 13]. However, the emotional content of the current face stimuli has been previously validated [12, 13] in a population which is geographically (Italy vs. South Africa), ethnically, and socioeconomically different from the current participants. Therefore, behavioral rating scores were obtained to ensure that the participants agreed with the emotional valence and arousal previously attributed to the stimuli. Participants were asked to judge the emotional valence of the face stimuli on a scale from 1 to 3, where 1 = positive (happy), 2 = neutral, and 3 = negative (sad) emotion. The rating scores varied according to the intended emotional category (i.e., SP, MP, MN, SN) of the stimuli [*F*(1, 24) = 2405.73, *p* < .001, *partial η* ^*2*^ = .99] in a subset of randomly selected 25 participants. The rating scores increased monotonically between SP, MP, and MN (*p*s < .001) and reached a plateau at MN vs. SN (*p* = .37). In effect, SP was rated as positive (*M* = 1.0, SD = .04), MP close to neutral (*M* = 1.8, SD = .1), and both MN and SN as negative (*M*s > 2.9, SDs < .2).

In order to reduce the pupillary light reflex [15] elicited by the face stimuli, a visual non-face pattern was generated to be shown during the “pre-stimulus” interval before each face stimulus. This visual stimulus was produced by randomly permuting and then averaging the pixels derived from the entire set of face stimuli in order to match its grayscale intensity to that of the faces.

### Procedure

The participants sat in a dimly lit room with no external light other than that laminating from the screen. Before data acquisition, eye-tracking calibration was performed for each participant by requiring the participant to fixate on five targets. In instances where the gaze was not found at these targets, the calibration-procedure was repeated. The calibration results from a subgroup of participants are shown in Additional file 1: Figure S2.

**Fig. 2:**
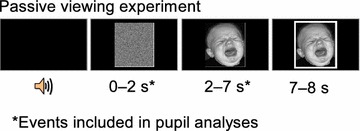
Trial structure in the experimental paradigm used to examine pupillary responses to infant facial expressions. Trial events consisted of (1) a *black screen* (duration = 1000 ms, a sound alarm was presented 500 ms prior to the next visual stimulus), (2) pre-stimulus display consisting of randomized pixels from the face images with stimulus-matched luminance (duration = 2000 ms), (3) face stimulus (duration = 5000 ms), and (4) a *white border* added to the face stimulus to signal the end of the trial. Pupil data were analyzed from the period starting from the pre-stimulus interval (displaying randomized pixels) and ending 5 s after the onset of the face stimulus

After calibration, participants were presented with a series of trials each consisting of (1) a short 1000-ms foreperiod with a black screen, (2) a 2000-ms pre-stimulus interval with a random visual pattern, (3) a 5000-ms face stimulus, and (4) a white rectangular border surrounding the face for 1000 ms to signal the end of the trial. A short sound signal, a “notification beep”, was presented 2500 ms before each face stimulus. Illustration of the stimulus sequence is shown in Fig. 2. The face stimulus for a given trial was selected randomly, without replacement, from the set of 36 faces (9/category as explained above). The stimuli were presented on a black background. The participants were asked to simply view each stimulus presented without a specific requirement for a response. To collect subjective ratings, the experimenter presented the face pictures to participants as paper print-outs and collected verbal responses from participants onto separate sheets after the entire sequence of pupil data acquisition.

### Acquisition and analysis of pupil diameter data

Pupil size was measured with a Tobii X-60 or X2-60 eye-tracker camera which measures corneal reflection of infrared light relative to the image of the pupil. The acquisition was controlled by custom-written MATLAB scripts, Psychtoolbox, and the Talk2Tobii toolbox, interfacing with a Tobii (Danderdyn, Sweden) eye-tracker.

**Fig. 3:**
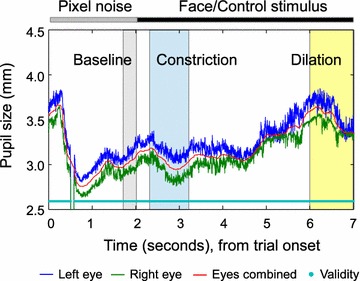
Single-trial pupil response. Pupil diameter as a function of time is shown from both eyes separately (*blue* = *left*, *green* = *right*) and from data combined across eyes (*red*). Valid data frames are indicated by *cyan* points. Pupil size during invalid data frames were replaced by interpolation in the pupil size combined across eyes (*red*). The first 2 s of each trial displayed a random visual pattern followed by a face stimulus (2–7 s). Pupil dilation was extracted as the mean pupil size during the time-window ranging from 4000 to 5000 ms after face onset (6000–7000 ms after trial onset, *yellow* background). Pupil constriction was extracted as the minimum pupil size from time window spanning 300–1200 ms after face onset (*blue* background). A baseline for pupil size was calculated from −300 to 0 ms relative to face onset (*gray* background)

Pupil size was measured from both eyes during the presentation of the face stimuli as well as during the pre-stimulus interval (with random visual pattern). The pupil data was acquired in conjunction with synchronous point-of-gaze (POG), eye-tracking validity (i.e., “valid” or “invalid”), and stimulus timing data at a sampling rate of 60 Hz. The data on pupil size was preprocessed using gazeAnalysisLib [24] to complete the following steps: averaging the diameter across the left and the right eye, replacing “invalid” frames of pupil size by the means of linear interpolation, median filtering, and baseline correction. The POG data was also combined across eyes and median filtered. The window size of the median filter was 7 frames (ca. 120 ms) for both POG and pupil data. A typical pupil response from a single trial is shown in Fig. 3.

**Fig. 4:**
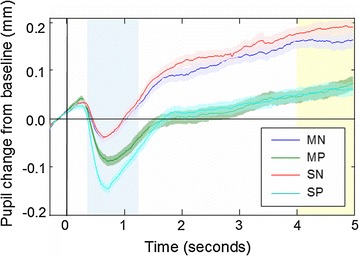
Grand average pupil response. Pupil time-series were baseline-corrected to the 300-ms interval preceding the face stimulus. The pupil response is shown for mild negative (MN) and positive (MP) as well as for strong negative (SN) and positive (SP) stimulus conditions. Face onset is indicated by the *vertical line* and the time-windows for pupil constriction and dilation by *blue* and *yellow* background, respectively. The *shaded* are around *curves* indicate standard error of the mean

Pupil response was baseline-corrected by subtracting the pupil size from a 300-ms pre-face time window. Based on visual inspection of the grand average pupil response, the minimum and maximum pupil diameters were reached at 300–1200 and 4000–5000 ms after face onset, respectively (Fig. 4). These time intervals were thus selected for the extraction of pupil constriction and dilation, respectively. While pupil constriction was determined as the minimum pupil size during the 300–1200 ms time window, pupil dilation was extracted as the mean pupil size during the latter 4000–5000 ms time-window.

Quality control (QC) of trial-by-trial pupil data was based on the following factors: (1) participant maintaining gaze within the face stimulus, (2) error-free pupil/eye-tracking, and (3) absence of outlier values. Quantitative indices of the participant gaze coordinates and error-free pupil tracking were acquired on a frame-by-frame basis together with pupil diameter. Using these metrics, trials where participant’s gaze was directed at the location of the face stimulus less than 10% of time, either in the baseline or in the response interval, were rejected from analysis. Similarly, trials with excess of eye/pupil-tracking frames labeled as “invalid” by the acquisition software were discarded from the analyses. These trials were defined as those where the longest streaks of consecutive non-valid frames exceeded 250 ms within the baseline period, or 900 ms within the time-window used for extracting the pupil constriction or dilation response. In addition to these basic QC measures, we identified the first trial within each measurement as a systematic source of outlier data, and rejected these trials from further analyses as well (see Additional file 1 for further details about the rejection criteria used in the study). On the average 6.2 (SD = 6.1) trials were rejected (out of 35 available) from each participant (averaged across constriction and dilation). Two participants had less than 3 averaged trials available for the analysis of the effects of Arousal and Valence on pupil response, and were thus rejected from final statistical analyses.

The quality of the trials accepted for further analyses was then inspected by analyzing the average values of frame-by-frame pupil/eye-tracking data within these trials. The targeted metrics included the average percentage of valid frames for both eyes (during the response time-window), the duration of non-valid streaks during the baseline and response time-windows, and the percentage of gaze directed at the location of the face (minimum percentage across baseline and response time-windows). These statistics are shown in Table 1. In summary, mean valid eye-tracking reached 87–91%, and on the average, longest-non valid data streaks were shorter than 76–121 ms.

**Table 1:** Eye-tracking quality

	Constriction		Dilation	
	Mean	SD	Mean	SD
Valid eye-tracking (%)	90.5	17.6	87.0	19.8
Longest non-valid streak				
Baseline (ms)	28.5	61.0	27.9	60.5
Response (ms)	75.1	141.7	120.3	176.9
Inside AOI, valid frames (%)	96.1	21.6	95.4	23.5

### Statistical analyses

In order to determine whether significant pupil constriction and dilation were elicited by the current stimuli, baseline-corrected pupil diameter was contrasted to the zero-level (i.e., no response) with one sample *t*-tests. The emotive pupil response, in turn, was defined as a change in pupil size between conditions differing in emotive valence or arousal of the stimuli. Emotion-related differences in baseline-corrected pupil diameter across stimulus conditions and response time-windows were investigated with a Time-window (2) × Arousal (2) × Valence (2) repeated-measures Analysis of Variance (ANOVA). The factors comprised constriction vs. dilation phase (Time-window), strong vs. mild emotion (Arousal), and negative vs. positive emotion (Valence), respectively. Both main an interaction effects were analyzed. In case interaction effects were found in the initial ANOVA, further comparisons within pupil constriction and dilation data, separately, were conducted with Arousal × Valence ANOVAs. Any subsequent pairwise tests were conducted with pair-wise *t*-tests. Effect sizes are reported using Cohen’s *d* for *t*-tests and *partial η* ^2^ for ANOVA throughout the results.

## Results

Following previous research [15–20], the presentation of faces was expected to elicit a typical visually induced pupil response consisting of an initial pupil constriction (decrease in pupil diameter *Ø*) and a subsequent dilation (increase in pupil diameter *Ø*). Further, as an increase in pupil size has been documented for pictures (not always including faces) with strong vs. mild emotive arousal [15, 17, 18], the pupil size was hypothesized to be larger both during the constriction and the dilation phase for strong vs. mild expressions. The corresponding effects were investigated also in response to negative vs. positive emotional valence of the facial expressions.

An ample pupil constriction, that is, a decrease in pupil diameter during 300–1200 ms after stimulus onset (Fig. 4) against baseline level, was found in response to all face stimuli [|*ΔØ*| > .11 mm, |*ts*| > 13.18, *p*s < .001, |*d*s| > 1.43]. Pupil constriction was followed by a subsequent pupil dilation (Fig. 4) and the pupil size increased significantly above the pre-stimulus baseline during the latter 4000–5000 ms time window [.05 < ΔØ < .19 mm, *t*s > 3.96, *p*s < .001, .43 < |*d*s| < 1.44].

Main effects of Time-window [constriction *vs*. dilation; *F*(1, 83) = 532.40, *p* < .001, *partial η* ^2^ = .87], and Valence [*F*(1, 83) = 157.03, *p* < .001, *partial η* ^2^ = .65] were found on baseline-corrected pupil size. These effects were due to greater pupil size during dilation (*ΔØ* = .12 mm) vs. constriction (*ΔØ* = −.16 mm) and greater pupil size in response to negatively-valenced vs. positively-valenced stimuli. Interaction effects on pupil size were found between Arousal and Valence [*F*(1, 83) = 7.76, *p* < .01], Time-window and Valence [*F*(1, 83) = 15.03, *p* < .001], as well as Time-window and Arousal [*F*(1, 83) = 13.33, *p* < .001]. Therefore, the effects of Arousal and Valence on pupil size were inspected separately for pupil constriction and dilation.

Main effects of Valence [*F*(1, 84) = 83.60, *p* < .001, *partial η* ^2^ = .50] and Arousal [*F*(1, 84) = 14.22, *p* < .001, *partial η* ^2^ = *.15*] were found on pupil constriction. Contrary to the hypothesis of increased pupil size in response to stimulus arousal, strong stimulus arousal was related to a decreased pupil size (i.e., increased constriction) within this early time window [strong < mild; *ΔØ* = −.03 mm]. Moreover, there was an interaction between Valence and Arousal on pupil constriction [*F*(1, 84) = 11.64, *p* < .001], reflecting a significant effect of arousal for the positively-valenced stimuli [*ΔØ* = −.05 mm, *t*(84) = −4.77, *p* < .001, *d* = −.52] but not for the negatively-valenced stimuli [*ΔØ* = −.01 mm, *t*(84) = −.99, *p* = .33, *d* = −.11]. While the hypothesized emotion-related increase in pupil diameter during constriction was not found for stimulus arousal, decreased pupil constriction (i.e., greater pupil diameter), was found for faces with negative *vs*. positive emotional valence across both levels of arousal [*ΔØs* > .05 mm, *ts*(84) > 6.10, *ps* < .001, |*d*|s > .66].

A main effect of Valence [*F*(1, 83) = 129.31, *p* < .001, *partial η* ^2^ = .61] was found on pupil dilation due to a .12-mm increase in pupil size to negatively-valenced (*ΔØ* = .17) vs. positively-valenced infant facial expressions (*ΔØ* = .06). Contrary to the hypothesized arousal-related pupil dilation, no effect of arousal on pupil dilation was found [*F*(1, 83) = 1.41, *p* = .24]. No interaction between Arousal and Valence [*F*(1, 83) = 2.54, *p* = .12] was found on pupil dilation either.

## Experiment 2

### Methods

#### Participants

The participants in Experiment 2 consisted of 15 volunteers (9 female, Age: *M* = 28.9 years, *range* = 24–50 years). None of the participants in Experiment 2 participated in Experiment 1 and parenthood was not required for the inclusion of participants into Experiment 2. The study was ethically approved by the institutional review board of the University of Stellenbosch and written informed consent was obtained from all participants.

#### Stimuli

**Fig. 5:**
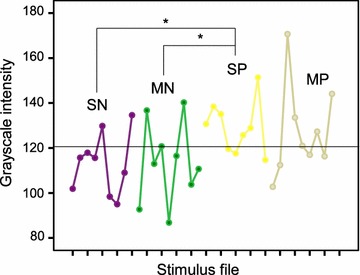
*Grayscale* (bitmap) intensity values of the stimuli. Pair-wise comparisons between means of different stimulus categories indicate higher intensity (and, hence, luminance) for face stimuli with “strong positive” vs. “negative” emotional expressions. *SN* strong negative, *MN* mild negative, *SP* strong positive, *MP* mild positive. *Horizontal line* indicates the mean *grayscale* intensity across all stimuli

The stimuli in the original pool of infant facial expressions were matched for luminance [12], but as pupil diameter is highly sensitive to stimulus luminance, a further analysis of brightness was conducted for the purposes of the present study. Optical luminance data were unavailable (requires careful photometric measurements with appropriate equipment), but the possible differences in luminance were inspected from mean grayscale intensity (0–255) values of the bitmap files. A one-way ANOVA showed that, overall, there were no statistically significant differences between stimulus categories in grayscale intensity values, [*F*(4,32) = 1.92, *p* = .13, *partial η* ^2^ = .19], but inspection of the bitmap intensity for individual images and direct pairwise comparisons showed noticeable variation within and across stimulus categories in intensity (Fig. 5). Furthermore, while the random visual pattern presented before each face stimulus had grayscale intensity equal to the mean intensity across *all faces* (horizontal line in Fig. 2), it differed somewhat in intensity from *each individual face* stimulus. Because such differences in stimulus luminance might bias the emotional effects on pupil response, we generated an array of control stimuli by randomly scrambling the bitmap matrices of the face images. The resultant control stimuli, thus, had exactly the same mean grayscale intensity as the face stimuli. These non-face stimuli were presented to a group of new participants (*N* = 15) who also viewed the original face pictures used in Experiment 1. The same visual pattern, consisting of randomly permuted pixels, was presented during the pre-stimulus interval as in Experiment 1.

### Procedure

The stimulation paradigm was identical to that in Experiment 1 (Fig. 2). In particular, the sequence and timing of events within experimental trials was similar across the experiments. However, an additional experimental condition was included where the face stimuli were replaced by their pixel-scrambled counterparts. That is, in addition to face stimuli, random non-face patterns were presented in a separate experimental block preceding or following the face sequence in a counterbalanced order across participants.

### Acquisition and analysis of pupil diameter data

The same equipment, software, and parameters were used in the acquisition of the pupil data as in Experiment 1. The pupil constriction and dilation were likewise extracted from the same time-windows spanning 300–1200 and 4000–5000 ms after face onset. An average of 6.0 (SD = 6.5) and 5.5 (SD = 7.6) trials were rejected per participant in the face and the control (pixel-scrambled face) condition, respectively. However, all participants had a sufficient number of averaged trials (≥3) for the statistical analyses of the effects of Arousal and Valence. Thus data from all participants were included in the final statistical analyses from Experiment 2.

### Statistical analyses

Pupil change from baseline (zero-level) in all stimulus conditions separately was first analyzed with one-sample *t*-tests. Then, emotion-related differences in baseline-corrected pupil diameter across stimulus conditions and response time-windows were compared with a Pixel randomization (2) × Time-window (2) × Arousal (2) × Valence (2) repeated-measures ANOVA. The repeated-measures factors comprised intact faces vs. scrambled non-faces (Pixel randomization), constriction vs. dilation phase (Time-window), strong vs. mild emotion (Arousal), and negative vs. positive emotion (Valence), respectively. Both main an interaction effects were analyzed. In case interaction effects were found in the initial ANOVA, further comparisons within pupil constriction and dilation data, separately, were conducted with Pixel randomization × Arousal × Valence ANOVAs. Finally, if an interaction was found between emotional pupil response (i.e., Arousal or Valence) and Pixel randomization, Arousal × Valence ANOVAs were conducted separately for the face and the-noise condition to qualify the source of the interaction effect. Effect sizes are reported as *partial η* ^2^.

## Results

Pupil constriction was elicited across all conditions (|*ΔØ*| > .08 mm, |*t*s| > 4.19, *p*s < .001, |*d*s| > 1.08) including both those with face stimuli and those with the non-face stimuli (randomly permuted pixels). However, significant pupil dilation above pre-stimulus baseline was observed only in the face condition (*ΔØ* > .10 mm, 2.24 < *t*s < 5.16, *p*s < .05, .58 < *d*s < 1.33). Such pupil dilation was invariantly absent in the non-face condition (*ΔØ* ≤ .06 mm, −1.24 < *t*s < 2.01, *p*s > .06, −.32 < *d*s < .52).

Main effects of Time-window [*F*(1, 14) = 80.57, *p* < .001, *partial η* ^2^ = .85], Pixel randomization [*F*(1, 14) = 13.05, *p* < .01, *partial η* ^2^ = .48], and Valence [*F*(1, 14) = 51.22, *p* < .001, *partial η* ^2^ = .79] were found on pupil size across all conditions within Experiment 2. However, interaction effects on pupil size were found between Time-window and Pixel randomization [*F*(1, 14) = 10.29, *p* < .01] and between Valence, Time-window, and Pixel randomization [*F*(1, 14) = 7.26, *p* < .05]. Therefore, the effects of Valence on pupil size were inspected separately from the two different time-windows (constriction and dilation), and further, separately within the face and the non-face condition. The purpose of these further analyses was to determine whether the effect of Valence on pupil size differed between the face condition, with genuine emotional signals, and the non-face condition, without emotional content. No main or interaction effects of Emotional arousal on pupil size were found.

A main effect of emotional Valence was found on pupil constriction [*F*(1, 14) = 41.93, *p* < .001, *partial η* ^2^ = .75]. A trend for interaction between Valence and Pixel randomization was further found on pupil constriction [*F*(1, 14) = 3.07, *p* < .11]. However, the effect of Valence was found both within the face condition [*F*(1, 14) = 12.77, *p* < .001, *partial η* ^2^ = .48] and within the non-face condition [*F*(1, 14) = 35.10, *p* < .001, *partial η* ^2^ = .72] in subsequent tests. As the effect of Valence on pupil constriction was found both in the face and in the non-face control condition, it cannot be considered to indicate an emotive pupil response (the non-face stimuli consisted of meaningless random patterns).

**Fig. 6:**
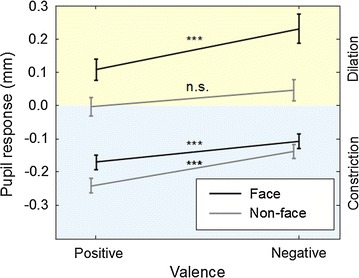
Pupil diameter during constriction (*lower half*) and dilation phase (*upper half*) in response to negative and positive facial expression of emotion and to luminance-matched non-face stimuli (*scrambled pixels*). Greater pupil dilation was elicited by negatively-valenced than by positively-valenced faces. No such difference in dilation was found across non-face stimuli. While the effect of valence on pupil size was found also during the constriction phase, this effect was not unique to face stimuli. Data from the control experiment (Experiment 2, *N* = 15). *Error bars* indicate standard errors of the mean. ***p < .001, *n.s*. not significant

A main effect of emotional Valence was also found on pupil dilation [*F*(1, 14) = 17.85, *p* < .001, *partial η* ^2^ = .56]. Furthermore, a trend level interaction [*F*(1, 14) = 3.80, *p* < .08] was found between Valence and Pixel randomization on pupil dilation (Fig. 6). This interaction was qualified by a significant effect of Valence on pupil dilation in the face condition [*F*(1, 14) = 23.30, *p* < .001, *partial η* ^2^ = .63] but not in the non-face condition [*F*(1, 14) = 2.78, *p* < .12, *partial η* ^2^ = .17]. In the face condition, greater pupil dilation was found for negatively-valenced (*ΔØ* = .23 mm) than for positively-valenced (*ΔØ* = .11 mm) infant face stimuli (difference = .12 mm).

As the non-face stimuli, consisting of random pixels, may be less motivating for participants to attend to than faces, it was necessary to ensure that the participants fixated equally on both stimulus types. Equal participant attentiveness and data quality in face and non-face condition was indicted by invariable number of acceptable trials across conditions [*F*(1, 14) = .14, *p* = .72, *partial η* ^2^ = .01]. Therefore, the difference between face and non-face condition reflect true effects of face and emotion processing as verified against conceivable data quality issues.

## Discussion

Pupillary response, in particular pupil dilation, has been proposed as an indicator of variable psychophysiological states [14, 15, 20]. In the current study, we investigated whether the emotive pupil response could be used to index mothers’ responsiveness to infant non-verbal communication. To this end, in Experiment 1, we measured pupillary responses in mothers while they viewed infant facial expressions. Larger pupil dilation was evoked by infant signals of distress or discomfort than by positively-valenced facial expressions. Emotive pupil dilation was further replicated in comparison against a non-face control condition in Experiment 2, a separate control experiment. The control experiment further showed that the pupil dilation response triggered by infant distress was dissociable from a response to the brightness of stimuli in this category.

In child and adolescent participants, pupil dilation has been previously reported in response to face stimuli with direct as opposed to averted gaze, especially when depicting happy facial expressions [18]. In contrast, greater pupil dilation in response to angry vs. happy or fearful facial expressions has been reported in adult participants [31, 32]. Furthermore, the sensitivity of the pupil response to direct vs. averted gaze found in typically developing participants was absent in children diagnosed with autism spectrum disorders [18]. Therefore, the sensitivity of the pupil response to facial expressions of emotion seems to depend on participant population, stimulus material, or on their combination [18, 31, 32]. Consequently, previous literature on emotive pupil response is insufficient to describe the perception and physiological responsiveness to infant facial expressions in mothers or to describe how such processes are reflected in the pupil response. The current study (Experiment 1) is the first pupillometric study using specifically infant facial expressions of emotion as stimuli and mothers of young infants as participants. The current results characterize the pupil response in this particular context and indicate increased autonomic responsiveness in response to infant signals of discomfort and distress.

Adaptive infant-caregiver interaction rests on the ability of the interactants to receive and express emotional signals through facial expressions [1]. Mutually positive, optimally arousing social interaction involves the regulation of the activity of the autonomic nervous system as a component of emotion regulation [25] and correlates of maternal sensitivity have been found in both sympathetic and parasympathetic activity. Activation of the sympathetic nervous system is associated with emotional arousal and has been previously indicated in mothers’ response to infant cry as indexed by electrodermal measures [26, 27]. Despite wide psychophysiological application [20, 28–30], relatively few studies have used pupillometry to investigate emotional processes evoked by the perception of facial expressions [18, 31, 32]. Such recent studies, using adult faces as stimuli, have indicated greater pupil dilation in response to angry vs. happy or fearful facial expressions in adult participants [31, 32]. In the current study, we investigated specifically whether the emotional responsiveness in mothers to infant facial expressions might be indexed with pupillometry. Our results were consistent with the previous studies on pupillary responses to facial expressions of emotion by indicating an increase in baseline-corrected pupil size in response to emotional face stimuli. Importantly, this effect was now replicated in the special case of infant stimuli viewed by mothers.

While autonomic responsiveness may be a prerequisite for adequate mother-infant interaction, overactive sympathetic arousal to infant or child cues has been linked with harsh parenting [26], lower maternal sensitivity [27], negative appraisal of children [33], and child abuse [34]. Also parasympathetic activity reflected in the respiratory sinus arrhythmia, in both baseline level [35] and in the regulation of the vagal tone [36], has been linked to maternal sensitivity. Moreover, the sympathetic and parasympathetic systems may act in concert in determining emotional response in mothers to infant crying and distress [27]. In the current study, emotive response to infant faces was established in pupil dilation which has been associated with sympathetic activity [16]. In this light, the current emotive pupil response is analogous to the increased skin conductance in mothers elicited by sounds of infant cry, which is also attributed to sympathetic arousal [26, 27]. This interpretation is further supported by covariance between emotive pupil dilation and skin conductance in response to affective pictures [15]. However, the pupil size during dilation may reflect the level of parasympathetic activity as well [16]. Based on findings from autonomic responses to infant cry [26, 27], the pupil response might index either sufficient or excessive autonomic arousal to infant negative affect for the maintenance of adaptive maternal sensitivity. In future studies, mapping the maternal pupil response to favorable level of autonomic responsiveness to infant cues might be achieved by relating the response to indices of caregiver behaviors and maternal sensitivity.

The norepinephrine attentional system of the brain originating in the locus coeruleus (i.e., the LC-NE system) has been suggested to underlie emotional pupil dilation [20, 37, 38]. Therefore, maternal pupil dilation in response to infant negative facial expressions is likely to share some common mechanisms with emotional pupil dilation in general which is elicited by a wide range of stimuli and conditions [20, 28–30]. Yet, there is evidence that effects of social signals of emotion on pupil size may reflect distinct social-cognitive processes. Firstly, interpersonal mimicry of gestures including mimicry of the pupil size [39] may specifically modulate the pupil response to faces. Secondly, previous studies suggest that there may be a dissociable neurocognitive system involved in monitoring infants’ emotional cues which is important for supporting parental caregiving [2, 12, 13]. Thus, while probably mediated by the attentional LC-NE system, the current results may be viewed as indexing a specific subcategory of social cognition related to face-to-face interaction and caregiving behaviors.

Given inter-individual variability in the accuracy to interpret infant facial expressions [13], we used a behavioral rating task to assess recognition of infant emotional signals in the current participants. The results from the rating task indicated high accuracy in the recognition of infant facial expressions. In our pupillary analyses, a distinction between stimuli rated as indicating negative emotion produced a pupil dilation which was larger than that elicited by stimuli rated as positive or neutral. Thus, the pupil response was associated with the subjective identification of negative vs. positive affect in the infant pictures. In future studies, a comparable approach combining pupillometry and behavioral performance could be used in studies involving specific participant groups with variable social cognitive abilities especially related to infant signals of emotion (e.g., from families at-risk for maladaptive infant-caregiver interaction).

Previous research has demonstrated an effect of emotional arousal rather than that of valence on pupil dilation [15]. In contrast, in the current study we found an effect of emotional valence on pupil dilation but no effect of stimulus arousal. The difference between the findings may be related to the type of stimuli (face vs. IAPS, not limited to faces), the type of people depicted (infants *vs*. IAPS, not limited to infants), and the scale used in stimulus classification. Perceptual scaling of any stimulus is inherently arbitrary and heavily influenced by the reference stimulus or stimuli [40, 41]. In scaling emotional valence and arousal different sets of stimuli, and hence different reference(s), may have been used in the current stimulus set in comparison to IAPS pictures [42]. Thus, the arousal and valence categories used here may be different from those used in the IAPS. It further seems possible that the negatively-valenced stimuli in the current study depicting infant distress or discomfort may signal (and elicit) stronger emotional arousal than the positively-valenced faces used in the current study. That is, the dimensions of arousal and valence are not orthogonal as difference in valence between stimuli requires a sufficient level of arousal to emerge [42].

In principle, the onset and the time course of pupil dilation following an emotional stimulus could be estimated from the latencies of the LC-NE subsystems and their influence on pupil size [20]. To our knowledge, such estimates of the time course of the pupil response have not been established. In practice, the emotive pupil response has been investigated from different time-windows spanning 500–1300 ms [17], 600–1600 ms [18], 1000–1300 ms [43], 2–4 s [31, 32], or 2–6 s [15, 17] after stimulus onset. The early and late time-windows have been typically selected to cover the constriction and the dilation phase, respectively. In the current study, a relatively late time-window spanning 4–5 s after stimulus onset was chosen for the extraction of pupil dilation in order to minimize the contribution of the pupillary light response on the estimate. The current results indicate that the pupil dilation within this time-window was both sensitive to the emotive content of the stimuli and independent of stimulus brightness. In future studies using constant light conditions, a more detailed, frame-by-frame, analysis of the pupil response together with known latencies of the LC-NE system [20] might provide insight into the time course of LC activation in the context of emotional perception.

Pupillary response elicited by emotive face stimuli already around 600–1600 ms after stimulus onset in child participants [18], have been observed in previous studies. This latency overlaps with the pupil constriction reflex extracted in the current study. Furthermore, while a study [15] using pictures from the International Affective Picture System (IAPS), found no evidence for emotional effects in pupil constriction, a more recent study from the same authors indicated emotional suppression of this initial light reflex [17]. In the current study, modulations of pupil constriction in response to stimulus category were found across both experiments. However, the control experiment (Experiment 2) indicated that these effects were not specific to emotional content or to faces as they were found for the random non-face stimuli as well. The difference between the current results from those obtained with IAPS [17] may be related to the type of emotionally salient stimuli used to evoke the autonomic pupil response: in the IAPS study, the largest emotional suppression of pupil constriction was found for erotic and violent scenes which were not used in the current study but may elicit CNS [44] and ANS [45] activity distinct from other emotionally equally arousing stimuli. Further, the pupil constriction in the current study may have been partially suppressed by the presentation of the pre-stimulus visual pattern, which might also have suppressed emotional effects on this initial light reflex. Thus, further studies may be needed to clarify the modulations of pupil constriction by affective face processing, especially in the context of maternal responses to infant emotive cues.

Pupillary responses to emotional cues are relatively small and intermixed with the larger effects of stimulus or ambient luminance. In the current study, stimuli in different emotion categories were not significantly different with respect to their mean (bitmap) intensity values, but there was a clear trend for both within- and between-category variability. An ideal solution for avoiding these confounds in emotion research would be to use stimuli with invariable luminance levels as well as matched contrast and spatial frequency profile [46]. However, in many cases perfect matching between stimulus luminance and other low-level features may be difficult or result in unnatural stimulus qualities. In the current study, confounding effects of stimulus luminance (i.e., variable face luminance and the luminance variability between the stimulus and the pre-stimulus intervals) were controlled by contrasting pupil responses elicited by face stimuli to those elicited by pixel-scrambled version of the same face stimuli. In this control experiment (Experiment 2), the stimulus light intensity was exactly matched to the face condition while all facial and emotive cues were removed from the stimuli. If the difference in pupil size across stimulus conditions were to persist in the control condition, such effects could simply be attributed to differences in stimulus luminance. Conversely, if the effects are unique to the face condition, they most likely stem from genuine emotive processes related to face perception. While pupil constriction was affected by stimulus category in both face and control condition, pupil dilation and it’s modulation by emotive stimulus category were confined to the face condition only. Thus, we may confidently interpret the current results as indicating emotive pupil dilation elicited by infant faces which is further intensified by negative emotional expressions.

## Limitations of the study

(1) The participants were not perfectly matched across the main and the control experiment. For example, unlike in the main experiment, the participants in the control experiment were both male and female, and parenthood was not required as an inclusion criterion. However, participants in both experiments were healthy adults and manifested very similar pupil dilation in response to stimuli depicting infant distress or discomfort. (2) The infants depicted in face stimuli were Caucasian while the participants viewing the stimuli where both Black and Caucasian, with low and high SES, respectively. Therefore, own-race biases in face processing [47] and SES [48, 49] might have affected the emotive responses elicited by the stimuli. (3) The current study focused in testing intra-individual variation in pupil response across variable infant facial expressions. Therefore, measures of inter-individual variations in potentially related variables such as maternal sensitivity to infant cues were not presented. As such, positive effect of increased pupil size in response to pictures of infant negative affect was found within the current sample consisting of healthy mothers (main experiment) and adult controls (control experiment). Future studies are needed to indicate whether the pupil response to infant faces is sensitive to inter-individual variations in general and in relation to motherhood in particular.

## Conclusions

Our current results indicate that pupil diameter is a sensitive marker of emotional processes elicited by infant facial expressions in the targeted participant group of mothers of infant children. While the perception of infant signals of distress may constitute a specific case of face processing [2, 12, 13], the current approach may be applicable to other domains of social perception as well due to a common psychophysiological pathway, the LC-NE system. Consequently, it remains possible that comparable pupil response may be elicited by non-infant stimuli as well or in non-mother viewers exposed to affective facial stimuli. In order to address the specificity of the current emotive pupil response to infant cues, further studies with both adult and infant face stimuli as well as participants with sufficient inter-individual variability in responsiveness to facial expressions of emotion in both categories are needed. Nevertheless, the principle contribution of the current study is in indicating the feasibility of pupil diameter as an index of mothers’ perception and responsiveness to infant non-verbal communication. As such, pupil diameter may provide a useful and accessible measure for studies of individual variations in mother-infant interaction [1].

## Additional file

**Additional file 1.** Additional figures.

## Abbreviations

ANOVA: analysis of variance
ANS: autonomic nervous system
AOI: area of interest
CNS: central nervous system
GLM: general linear model
IAPS: International Affective Picture System
LC-NE: locus coeruleus-norepinephrine
MN: mild negative
MP: mild positive
POG: point of gaze
SES: socioeconomic status
SN: strong negative
SP: strong positive

## References

[1] Strathearn L (2011). Maternal neglect: oxytocin, dopamine and the neurobiology of attachment. J Neuroendocrinol.

[2] Peltola MJ, Yrttiaho S, Puura K, Proverbio AM, Mononen N, Lehtimäki T (2014). Motherhood and oxytocin receptor genetic variation are associated with selective changes in electrocortical responses to infant facial expressions. Emotion.

[3] Rilling JK (2013). The neural and hormonal bases of human parental care. Neuropsychologia.

[4] Swain JE, Lorberbaum JP, Kose S, Strathearn L (2007). Brain basis of early parent-infant interactions: psychology, physiology, and in vivo functional neuroimaging studies. J Child Psychol Psychiatry.

[5] Yrttiaho S, Forssman L, Kaatiala J, Leppänen JM (2014). Developmental precursors of social brain networks: the emergence of attentional and cortical sensitivity to facial expressions in 5 to 7 months old infants. PLoS ONE.

[6] Peltola MJ, Forssman L, Puura K, van Ijzendoorn MH, Leppänen JM (2015). Attention to faces expressing negative emotion at 7 months predicts attachment security at 14 months. Child Dev.

[7] Barrett J, Fleming AS (2011). Annual research review: all mothers are not created equal: neural and psychobiological perspectives on mothering and the importance of individual differences. J Child Psychol Psychiatry.

[8] Glocker ML, Langleben DD, Ruparel K, Loughead JW, Gur RC, Sachser N (2009). Baby schema in infant faces induces cuteness perception and motivation for caretaking in adults. Ethology.

[9] Glocker ML, Langleben DD, Ruparel K, Loughead JW, Valdez JN, Griffin MD (2009). Baby schema modulates the brain reward system in nulliparous women. Proc Natl Acad Sci USA..

[10] Kringelbach ML, Lehtonen A, Squire S, Harvey AG, Craske MG, Holliday IE (2008). A specific and rapid neural signature for parental instinct. PLoS ONE.

[11] Proverbio AM, Riva F, Zani A, Martin E (2011). Is it a baby? Perceived age affects brain processing of faces differently in women and men. J Cogn Neurosci.

[12] Proverbio AM, Brignone V, Matarazzo S, Del Zotto M, Zani A (2006). Gender and parental status affect the visual cortical response to infant facial expression. Neuropsychologia.

[13] Proverbio AM, Matarazzo S, Brignone V, Del Zotto M, Zani A (2007). Processing valence and intensity of infant expressions: the roles of expertise and gender. Scand J Psychol.

[14] Beatty J, Lucero-Wagoner B, Cacioppo J, Tassinary LG, Berntson GG (2007). The Pupillary System. Handbook of psychophysiology.

[15] Bradley MM, Miccoli L, Escrig MA, Lang PJ (2008). The pupil as a measure of emotional arousal and autonomic activation. Psychophysiology.

[16] Steinhauer SR, Siegle GJ, Condray R, Pless M (2004). Sympathetic and parasympathetic innervation of pupillary dilation during sustained processing. Int J Psychophysiol.

[17] Henderson RR, Bradley MM, Lang PJ (2014). Modulation of the initial light reflex during affective picture viewing. Psychophysiology.

[18] Sepeta L, Tsuchiya N, Davies MS, Sigman M, Bookheimer SY, Dapretto M (2012). Abnormal social reward processing in autism as indexed by pupillary responses to happy faces. J Neurodev Disord..

[19] Prehn K, Kazzer P, Lischke A, Heinrichs M, Herpertz SC, Domes G (2013). Effects of intranasal oxytocin on pupil dilation indicate increased salience of socioaffective stimuli. Psychophysiology.

[20] Laeng B, Sirois S, Gredebäck G (2012). Pupillometry: a window to the preconscious?. Perspect Psychol Sci..

[21] Joshi S, Li Y, Kalwani RM, Gold JI (2016). Relationships between pupil diameter and neuronal activity in the locus coeruleus, colliculi, and cingulate cortex. Neuron.

[22] Loewenfeld I (1993). The pupil: anatomy, physiology, and clinical applications.

[23] Sheehan DV, Lecrubier Y, Sheehan KH, Amorim P, Janavs J, Weiller E (1998). The mini-international neuropsychiatric interview (M.I.N.I.): the development and validation of a structured diagnostic psychiatric interview for DSM-IV and ICD-10. J Clin Psychiatry..

[24] Leppänen JM, Forssman L, Kaatiala J, Yrttiaho S, Wass S (2015). Widely applicable MATLAB routines for automated analysis of saccadic reaction times. Behav Res Methods..

[25] Koole SL (2009). The psychology of emotion regulation: an integrative review. Cogn Emotion..

[26] Joosen KJ, Mesman J, Bakermans-Kranenburg MJ, van Ijzendoorn MH (2013). Maternal overreactive sympathetic nervous system responses to repeated infant crying predicts risk for impulsive harsh discipline of infants. Child Maltreat..

[27] Leerkes EM, Supple AJ, O’Brien M, Calkins SD, Haltigan JD, Wong MS (2015). Antecedents of maternal sensitivity during distressing tasks: integrating attachment, social information processing, and psychobiological perspectives. Child Dev.

[28] Laeng B, Orbo M, Holmlund T, Miozzo M (2011). Pupillary Stroop effects. Cogn Process.

[29] Dabbs JM (1997). Testosterone and pupillary response to auditory sexual stimuli. Physiol Behav.

[30] Cassady JM, Farley GR, Weinberger NM, Kitzes LM (1982). Pupillary activity measured by reflected infra-red light. Physiol Behav..

[31] Kret ME, Roelofs K, Stekelenburg JJ, de Gelder B (2013). Emotional signals from faces, bodies and scenes influence observers’ face expressions, fixations and pupil-size. Front Hum Neurosci..

[32] Kret ME, Stekelenburg JJ, Roelofs K, deGelder B (2013). Perception of face and body expressions using electromyography, pupillometry and gaze measures. Front Psychol..

[33] Lorber MF, O’Leary SG (2005). Mediated paths to over-reactive discipline: mothers’ experienced emotion, appraisals, and physiological responses. J Consult Clin Psychol.

[34] Frodi AM, Lamb ME (1980). Child abusers’ responses to infant smiles and cries. Child Dev.

[35] Musser ED, Ablow JC, Measelle JR (2012). Predicting maternal sensitivity: the roles of postnatal depressive symptoms and parasympathetic dysregulation. Infant Ment Health J..

[36] Moore GA, Hill-Soderlund AL, Propper CB, Calkins SD, Mills-Koonce WR, Cox MJ (2009). Mother-infant vagal regulation in the face-to-face still-face paradigm is moderated by maternal sensitivity. Child Dev.

[37] Aston-Jones G, Cohen JD (2005). An integrative theory of locus coeruleus-norepinephrine function: adaptive gain and optimal performance. Annu Rev Neurosci.

[38] Murphy PR, Robertson IH, Balsters JH, O’Connell RG (2011). Pupillometry and P3 index the locus coeruleus-noradrenergic arousal function in humans. Psychophysiology.

[39] Kret ME, Tomonaga M, Matsuzawa T (2014). Chimpanzees and humans mimic pupil-size of conspecifics. PLoS ONE.

[40] Kreiman J, Gerratt BR, Ito M (2007). When and why listeners disagree in voice quality assessment tasks. J Acoust Soc Am.

[41] Yrttiaho S, Alku P, May PJ, Tiitinen H (2009). Representation of the vocal roughness of aperiodic speech sounds in the auditory cortex. J Acoust Soc Am.

[42] Bradley MM, Lang PJ, Coan JA, Allen JB (2007). The international affective picture system (IAPS) in the study of emotion and attention. The handbook of emotion elicitation and assessment.

[43] Bradley MM, Lang PJ (2015). Memory, emotion, and pupil diameter: repetition of natural scenes. Psychophysiology.

[44] Schupp H, Cuthbert B, Bradley M, Hillman C, Hamm A, Lang P (2004). Brain processes in emotional perception: motivated attention. Cogn Emot.

[45] Lang PJ, Bradley MM, Cuthbert BN, Lang PJ, Simons RF, Balaban M (1997). Motivated attention: Affect, activation, and action. Attention and orienting.

[46] Link B, Junemann A, Rix R, Sembritzki O, Brenning A, Korth M (2006). Pupillographic measurements with pattern stimulation: the pupil’s response in normal subjects and first measurements in glaucoma patients. Invest Ophthalmol Vis Sci.

[47] Ekman P, Friesen WV, O’Sullivan M, Chan A, Diacoyanni-Tarlatzis I, Heider K (1987). Universals and cultural differences in the judgments of facial expression of emotion. J Pers Soc Psychol.

[48] Hackman DA, Gallop R, Evans GW, Farah MJ (2015). Socioeconomic status and executive function: developmental trajectories and mediation. Dev Sci..

[49] Kraus MW, Cote S, Keltner D (2010). Social class, contextualism, and empathic accuracy. Psychol Sci.

