# Supplementary material for: Mothers’ pupillary responses to infant facial expressions
Source: Behav Brain Funct. 2017 Feb 6;13:2. doi: 10.1186/s12993-017-0120-9 (PMC5292805; doi:10.1186/s12993-017-0120-9)
Supplement: Supplementary file 1 — Additional file 1. Additional figures. [file 12993_2017_120_MOESM1_ESM.docx]

**Supplementary material to article: Mothers’ pupillary responses to infant facial expressions**

Authors: Santeri Yrttiaho, Dana Niehaus, Eileen Thomas, and Jukka M. Leppänen

**Data quality control**

Quality control of pupil data from mother participants was based on eye-tracking validity during the analysis (constriction or dilation phase) and baseline windows. Statistical distribution (Figure S1) of the duration of breaks in valid eye-tracking data was used to find suitable criteria for accepting or rejecting trials for further analyses. The most frequent (57.8–70.8 %) class of trials contained only valid eye-tracking frames or the duration of non-valid streak was shorter than 100 ms during the response interval. However, a prominent class (7.3 % of trials) of bad trials was identified with complete loss of valid eye-tracking or with a duration of non-valid streaks exceeding 900 ms during the analysis time window. Trials belonging in this category were excluded from further analyses. Furthermore, a class of trials (15.0–18.0 % of trials across experiments) was found where the participant’s gaze coordinates were either completely missing due to invalid eye-tracking or they were outside the area of interest where the face stimulus was presented. This class of trials (largely overlapping with the previous) was likewise rejected from further analyses. Finally, pupil dilation in the first trial of the experiment was on the average larger than during the rest of the experiment (*ΔØ* = 0.17 mm, *F*(35, 4300) = 3.16, *p* < .001). Hence, pupil data from the first trial of each session was rejected from further analyses as well.


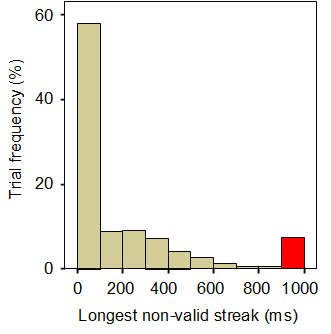


Figure S1. Longest non-valid streak in pupil dilation data. A class of trials with the longest streaks of bad eye-tracking frames (red bar) was rejected form further analyses.

While the primary ocular measure in the current study was pupil diameter, the point-of-gaze (POG) data was tracked simultaneously to ensure participants attendance to the face stimuli. The POG was calibrated before each experimental session and indicated good accuracy for all participants (Figure S2).


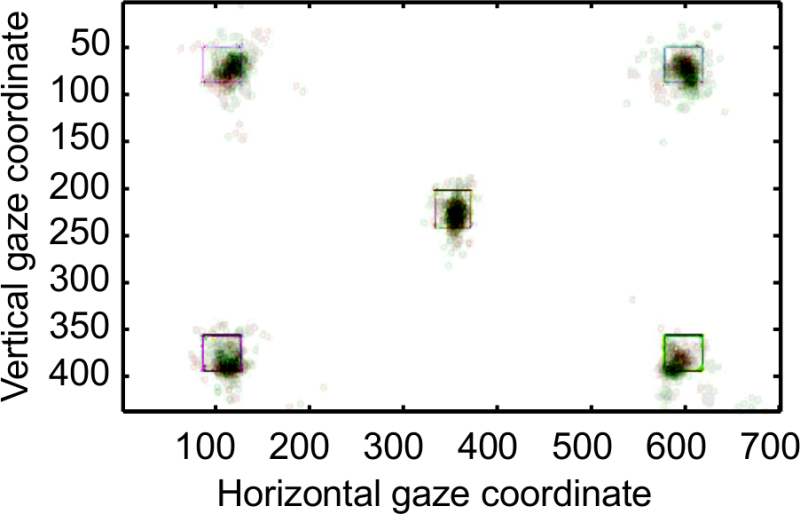


Figure S2. Calibration data across participants. Participants were asked to fixate at five targets (at the screen center and at its 4 corners) to ensure accurate and unbiased eye- and gaze-tracking in the beginning of each experimental session. The calibration outcome is exemplified by average gaze-coordinates from a subgroup of 21 participants.
